# An image‐guided technique for planning and verification of supine craniospinal irradiation

**DOI:** 10.1120/jacmp.v12i2.3310

**Published:** 2011-01-31

**Authors:** Ryan L. McMahon, Nicole A. Larrier, Q Jackie Wu

**Affiliations:** ^1^ Department of Radiation Oncology Duke University Medical Center Durham NC 27710 USA

**Keywords:** craniospinal, supine, image‐guidance

## Abstract

We present a technique for planning and verification of craniospinal treatment with the patient in the supine position. Treatment delivery and verification is streamlined through the use of modern imaging techniques. Treatments use two lateral brain fields abutted to a single or pair of posterior spine fields. Treatment delivery is simplified by aligning all isocenters in the anterior‐posterior and lateral directions. Patient positioning is accomplished via on‐board kV imaging. Verification of field shape and junctions is accomplished with BB placement and MV portal imaging. Daily treatment is simplified by using only longitundinal couch shifts, which are recorded in the patient chart and RV database. The technique is simple to implement in a clinic that is already using a similar beam arrangement with the patient prone. It requires no additional devices to be fabricated (for immobilization or QA), and it takes advantage of all the existing elements of a modern linac.

PACS number: 87.55.‐x

## I. INTRODUCTION

The goal of craniospinal irradiation (CSI) is to irradiate the entire neuraxis. This treatment is used for medulloblastoma and other brain tumors which tend to seed via transport in the cerebrospinal fluid. The classical setup for CSI includes two lateral brain fields abutted to one or two PA spine fields (depending on patient length). Collimator and couch rotation are typically used on the brain fields to bring their inferior border in alignment with the divergent superior border of the upper spine field. When two PA spine fields are used in this technique, they are not geometrically matched. Instead, the region of field overlap is simply placed anterior to the spinal cord. The patient is placed in the prone position to facilitate the direct visualization of light fields on the patient's skin. Since the spine is close to the skin, therapists and physicians depend on the field edge projections on the skin to confirm the proper field setup and junction. Furthermore, a gap is typically added to reduce the risk of overlap that would result from setup errors and/or patient motion. The gap locations are feathered by shifting their positions in the caudal‐cranial direction.

This classical setup has a number of limitations which have been the subject of numerous investigations. The dosimetry in the field junction with various gap sizes (including zero gap size) regions is well understood.^(^ ^1^ ^–^ ^3^ ^)^ These junctions introduce unwanted dose heterogeneity along the target volume. Novel field matching techniques and inter‐ or intrafractional junction shifts^(^ ^1^ ^–^ ^5^ ^)^ have been developed to improve dose uniformity and make CSI techniques more robust to setup uncertainties. The varying depth of the spinal cord along its length also introduces dose heterogeneity within the target volume. Simple compensation through single‐field intensity‐modulated radiation therapy (IMRT) has been employed to reduce this effect.^(^ ^6^ ^–^ ^7^ ^)^

However, the use of single PA spine fields for such intensity modulation results in substantial exit dose to the bowel and mediastinum. The effect of this exit dose is a concern in these cases, since a majority of patients receiving CSI are pediatrics. Multiple‐field IMRT^(^ ^8^ ^–^ ^9^ ^)^ or three‐dimensional conformal plans can reduce the magnitude of the exit dose at the expense of a larger low‐dose volume. Protons currently offer the best solution to the exit dose problem,^(^ ^6^ ^,^ ^10^ ^)^ but are not yet widely available. Though it isn't available in most clinics, tomotherapy also offers some unique advantages for CSI.^(11)^

Aside from the dosimetry concerns, the prone position is uncomfortable for most patients and impractical when anesthesia is needed. The greatly impaired mobility of many of these patients presents a difficulty for getting them into correct prone treatment position. Pediatric cases with anesthesia are not suitable for prone positioning, either. Many centers have developed techniques to deliver CSI with the patient in the supine position. This has resulted in a wide range of techniques appearing in the literature for both field setup and verification of field junctions.^(^ ^4^ ^,^ ^12^ ^–^ ^16^ ^)^ Here we report our technique for supine CSI treatment. In contrast to many previously published techniques, our emphasis was on (1) simplicity and efficient use of modern linac elements; and (2) making minimal changes to our protocol based on prone CSI treatment. Many aspects of CSI treatments (field junction verification, anesthesia, etc.) have the potential to extend treatment times, so simplicity is of utmost importance. Making minimal changes to our current protocol makes it easy to implement the technique into a clinical workflow, since the staff can build on prior knowledge and experience. These goals were accomplished by simplifying the setup of the three separate isocenters, and using modern imaging techniques for verification of field alignment.

## II. MATERIALS AND METHODS

### A. Simulation and immobilization

Patients are immobilized in the supine position with a thermoplastic head mask system that indexes directly to the treatment table (QFix Systems, WFT/Aquaplast Corporation, Avondale, PA). A customized thermoplastic headrest is made for pediatric patients for additional control of head rotation and chin extension. The chin is extended as much as possible without causing patient discomfort. When a disposable respiratory anesthesia mask is used, it is built directly into the thermoplastic mask. For patients not requiring anesthesia, wrist‐straps attached to a footboard are used to depress and immobilize the shoulders. A CT image is acquired from head to mid‐pelvis. For patients who will only receive craniospinal irradiation, 5 mm slice thickness is used. For patients who will have additional radiation (e.g., posterior fossa boost), 2.5 mm slice thickness may be used if the same image will be used for the entire treatment course.

### B. Treatment planning

**Figure 1:**
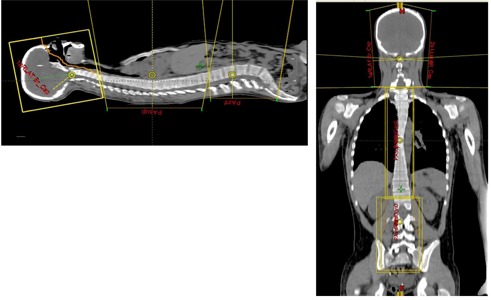
Illustration of isocenter alignment of multiple CSI fields. Isocenters of all fields share the same anterior‐posterior and lateral coordinates. Once the brain isocenter is chosen, the upper spine isocenter is shifted longitudinally by a multiple of 1 cm. The lower spine isocenter is then shifted from the upper spine by another multiple of 1 cm. This alignment requires only longitudinal couch shifts between isocenters for treatment.

Our technique uses the same field arrangement as the classical CSI technique (two lateral brain fields, 1–2 posterior spine fields), with modified isocenter placement to facilitate simple patient setup (Fig. 1). The isocenter of the brain fields is placed in the spine as inferiorly as possible in an effort to minimize the couch rotation. This isocenter then serves as a reference for the rest of treatment, and only a longitudinal shift is used for the spine field isocenters (i.e., only a couch longitudinal translation is necessary for treatment). This makes it important to straighten the patient during simulation, so that all isocenters are aligned with the spine. Because of different spine lengths, chin extensions and shoulder depressions, the longitudinal shifts will be patient‐specific. For simplicity and safety, the shifts are restricted to multiples of 1.0 cm.

At our facility, the driving factor for isocenter placement and field sizes is leaving enough room for the full range of gap shifts, as well as to minimize the use of couch rotation (if possible). Chin extension and shoulder compression also help to ensure enough room for multiple gap shifts. The collimator is rotated for the brain fields to match the divergence of the posterior upper spine field. A gap of 5 mm is used between the lateral brain fields and the PA upper spine. This gap is shifted superiorly by 5 mm every 9 Gy. When two spine fields are used, their geometric overlap is placed anterior to the spinal cord, and this gap is shifted inferiorly by 5 mm every 9 Gy.

### C. Treatment setup and field junction verification

#### C.1 Patient alignment verification with on‐board kV imaging

Daily treatment always starts by setting up to the isocenter of the opposed brain fields, which is used as a reference point for the entire fraction. For this task, we rely on kV imaging, which provides a high‐contrast image of the head and C‐spine. Comparing these images with the setup DRRs in orthogonal views (AP and RT) makes it easy to verify isocenter, chin extension and neck flexion. It also allows for quick assessment of the overall straightness of the patient. The remaining treatment fields are indexed to the brain fields through longitudinal couch shifts, so skin marks can serve as a simple verification of spine field alignment.

#### C.2 Individual field shape and junction verifications using on‐board MV imaging

On the first treatment day, or treatments following a gap shift, MV imaging is used to verify the field shape and gap junctions. The advantage of MV imaging is that it records the actual treatment field shape and position. Since the therapists are accustomed to obtaining visual verification of prone setups where PA spine fields can be directly verified on skin, our intention was to retain this ability in the new technique. However, since the treatment fields can no longer be directly projected on the skin, a new verification method had to be established.

**Figure 2:**
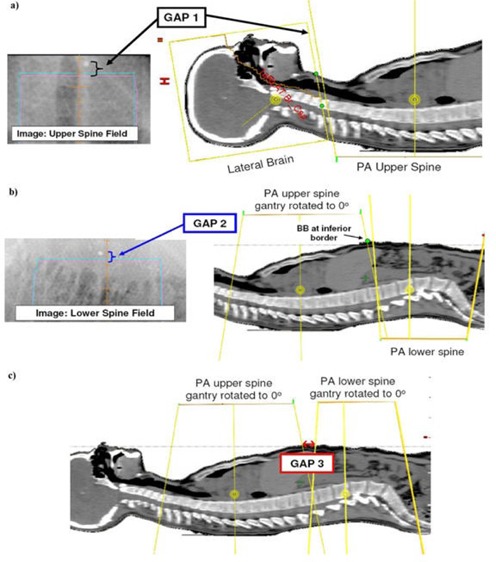
Illustration of gaps used to verify field junctions. Gap 1 represents the gap between the brain fields and upper spine. Gaps 2–3 are used to verify the abutment of the two spine fields. Gaps 1–2 (using BBs) are only verified on the first treatment day and on days after a gap shift has been performed. Gap 3 is verified on a daily basis.

For the purposes of verifying field junctions, three distances (gaps) are used:

**Gap 1** (Fig. 2(a)): **gap between brain fields and upper spine field.** This 5 mm gap is designed on the CT image. The actual gap is verified and documented by placing BBs at the inferior border of the brain fields before acquiring a portal image of the upper spine. The gap is measured as the distance between the BBs and the superior edge of the spine field in the portal image, and should agree with the planning field projections on the CT image (Fig. 2(a) right).

**Gap 2** (Fig. 2(b)): **gap 2 is used to verify and document the junction at the two PA fields.** The PA upper spine field is rotated to an AP orientation. A radio‐opaque marker (BB) is then placed on the patient's anterior surface at the inferior border of this light‐field projection. This BB is seen in a portal image of the lower spine (PA orientation). The distance seen in portal image between the BB and the superior border of the lower spine field is used as a reference to verify proper abutment of spine fields. This BB can appear inside or outside of field, depending on field size and patient thickness.

**Gap 3** (Fig. 2(c)): **the skin gap between the spine fields when they are rotated to AP orientations.** These light fields are marked on the patient's anterior surface and used to verify alignment on a daily basis. This gap is designed to replicate the visualization that is used for the field edge check on the skin for a prone treatment.

### D. Daily treatment flow

Each treatment day, orthogonal kV images are used to setup the patient to the isocenter of the brain fields. If it is the first treatment day, or the first day after a gap shift has been performed, then gaps 1–3 are all verified before delivering any treatment fields (i.e., all imaging is performed first).

On remaining days, kV imaging is still used to align the brain field isocenter, but only light‐field alignment is used to verify spine field alignment (through visualization of gap 3). Following the initial setup and treatment of the brain fields, the couch is simply translated longitudinally according to the shifts chosen in the treatment plan. A form is provided to the therapists to document the couch positions for each field in the patient chart. The couch position is also recorded in the treatment database. Since the patient is indexed to the table through the thermoplastic mask, the couch positions (following kV correction) should be similar for each fraction. Any couch position variation more than 5 mm requires a physics review. These recorded couch positions serve as second checks of isocenter alignment, in addition to the skin/mask marks.

All verification gaps are readily measured in the treatment planning system. Gap 2 should be measured in the imaging plane, rather than on the patient surface, though the numerical difference is very small over typical ranges of patient thickness and field sizes. The gaps are documented in the patient chart and provided to the therapists on a standardized form. This form is reviewed during the initial chart check, at which point the documented isocenter shifts and gap distances are checked against the treatment plan. During a weekly chart check, the form is reviewed to ensure that correct couch shifts are being applied at each treatment.

## III. RESULTS

Since implementing the new supine CSI technique in our clinic, seven patients have been treated, three of which required anesthesia. Patients ranged in age from 2–50 years. Total superior‐inferior extent of the treatment fields ranged from 51 cm to 85 cm, and four of the patients required two spine fields.

**Figure 3:**
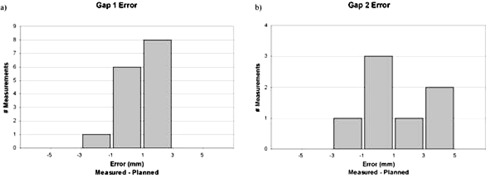
Distribution of measured gap errors for six patients treated with supine technique. Measurements done after planned couch shifts with BB placement described in Section II: a) gap 1: mean error=1.0 mm (σ=1.3 mm); b) gap 2: mean error = 1.6 mm (σ=2.1 mm).

Figure 3 shows the distribution of measured gap errors for gaps 1 and 2. Measurements were recorded on the first treatment day, and at each day following a gap shift. In all cases, patients were setup according to the brain isocenter, and gap measurements were done using the BB placement described above. The number recorded was the measured gap after the couch was translated longitudinally by the planned amount. The mean error for gap 1 was 1.0 mm, with a standard deviation of 1.3 mm. For gap 2, the mean error was 1.6 mm, with a standard deviation of 2.1 mm. Gap 3 was used as a redundant check of spine field alignment, and was not recorded on a daily basis.

## IV. DISCUSSION

Several methods for supine craniospinal irradiation have been presented in the literature,^(^ ^12^ ^–^ ^16^ ^)^ but many of these techniques were developed prior to the widespread introduction of advanced on‐board imaging tools. Thus, junction verification in these methods required offline phantom measurements^,^^(^ ^15^ ^)^ or a cumbersome apparatus for holding a piece of film.^(12)^ In at least one published technique, patient setup involved daily adjustment of fields according to light field projections.^(13)^ Another common limitation is that verification of field junctions often relies on gaps that are based on mathematical calculations.^(^ ^13^ ^,^ ^14^ ^,^ ^17^ ^)^ The unique feature of the technique presented here is that it takes advantage of online megavoltage (MV) and kilovoltage (kV) imaging tools to increase treatment efficiency and remove errors that may result from mathematically calculated gaps. MV portal imaging is ideally suited for verification of field borders and junctions for craniospinal irradiation, and integrated kV imaging with remote couch correction capability is well‐suited for evaluating patient setup. Another feature of this method that improves treatment efficiency is the alignment of isocenters in the lateral and vertical directions, although this has been previously discussed in the literature.^(15)^

### A. Simulation and immobilization

Our intention was to keep immobilization simple enough to accommodate a wide range of patients (pediatrics, adults, anesthesia, etc.). We rely on the combination of head fixation and a wrist‐strap/footboard system for full‐body immobilization. This could also be accomplished through the use of custom vacuum bags or other moldable body forms.

### B. Treatment planning

At our facility, the only modifications made to the treatment planning process were in the placement of isocenters and the documentation of verification gaps. Because of the importance of the longitudinal isocenter shifts, great care must be taken throughout the entire planning process to ensure that isocenters remained aligned in the AP and lateral directions.

Dosimetrically, this technique is similar to the classical CSI setup. As such, it suffers from dose nonuniformity, and produces substantial exit dose to the gut and mediastinum. Intensity modulation^(^ ^7^ ^–^ ^9^ ^)^ of spine fields could be easily incorporated into this technique to improve homogeneity. The dosimetry in the field junction regions is also similar to the classical CSI technique (since the same gap scheme is used). These gaps were put in place to serve as additional safeguards for setup errors and patient motions. This could be improved by novel field‐matching techniques and/or using intrafraction junction feathering.^(4)^ Since patient comfort improves from prone to supine and with improvements to the immobilization, we are confident that the intra‐ and interfraction reproducibility will improve over the prone method. With the support of image guidance, it should be feasible to reduce or eliminate field gaps and to implement other techniques that can improve dose heterogeneity in the target.

### C. Treatment verification

Since the patient is indexed to the table through the thermoplastic head fixation system, the couch positions after alignment of the brain isocenter should be similar each treatment day. We have not done a quantitative analysis of the expected range of variation, but have set a threshold of 5 mm difference at out facility. If initial couch positions differ by more than this amount along any axis, a physicist is asked to review the patient setup.

The indexing of the fields to each other (through longitudinal couch translation) provides a conceptual change in the analysis of field alignment. The fields can now be thought of as a single field, rather than three distinct isocenters. Because of this, our patients’ couch shifts are never modified as a result of discrepancies seen when measuring verification gaps. If the couch shifts are performed properly, this guarantees that the fields are an accurate representation of the treatment plan. Instead, any discrepancies in verification gaps are seen as a result of improper patient setup or BB placement. Thus, in this technique, we feel it is more robust to align the patient to the fields, rather than the fields to the patient. As such, the efficiency of this technique will be heavily dependent on the ability to properly immobilize the patient.

As described here, imaging is only used to setup the brain fields, so there is still the potential for misalignment of the spine fields. In our experience, careful alignment of anterior skin marks along the entire length of the treatment field has been sufficient for spine field alignment, but this may not be the case for all patients and institutions. Using on‐board imaging for the spine fields would reduce this uncertainty, but would add to setup time. Full‐body immobilization could also make setups more reproducible along the entire length of the patient.

## V. CONCLUSIONS

A simple technique for delivering craniospinal irradiation with the patient in the supine position has been developed. The technique is based on the classical CSI setup using two lateral brain fields and two PA spine fields. Treatment delivery is simplified by aligning all isocenters along the anterior‐posterior and lateral axes. A treatment verification technique was designed using a combination of BB placement and modern kV/MV imaging technology. Facilities which deliver CSI with the classical four‐field prone setup should be able to implement this supine technique with very few modifications to treatment planning procedures and treatment workflow.

## References

[1] Kiltie AE , Povall JM , Taylor RE . The need for the moving junction in craniospinal irradiation. Br J Radiol. 2000;73(870):650–54. 10911789 10.1259/bjr.73.870.10911789

[2] Cheng CW , Das IJ , Chen D‐J . Technical note: Dosimetry in the moving gap region in craniospinal irradiation. Br J Radiol. 1994;67(802):1017–22. 8000826 10.1259/0007-1285-67-802-1017

[3] Chang EL , Wong PF , Forster KM , Petru MD , Kowalski AV , Maor MH . Verification techniques and dose distribution for computed tomographic planned supine craniospinal radiation therapy. Med Dosim. 2003;28(2):127–31. 12804712 10.1016/S0958-3947(02)00248-0

[4] South M , Chiu JK , Teh BS , Bloch C , Schroeder TM , Paulino AC . Supine craniospinal irradiation using intrafractional junction shifts and field‐in‐field dose shaping: early experience at Methodist Hospital. Int J Rad Oncol Biol Phys. 2008;71(2):477–83. 10.1016/j.ijrobp.2007.10.029 18164864

[5] Koshy M , Paulino AC , Marcus RB , Ting J . The effect of an extended source‐to‐skin distance in the treatment of the spinal field in children receiving craniospinal irradiation. Med Dosim. 2004;29(1):7–10. 15023387 10.1016/j.meddos.2003.10.003

[6] St. Clair WH , Adams JA , Bues M , et al. Advantage of protons compared to conventional X‐ray or IMRT in the treatment of a pediatric patient with medulloblastoma. Int J Rad Oncol Biol Phys. 2004;58(3):727–34. 10.1016/S0360-3016(03)01574-8 14967427

[7] Pai Panandiker A , Ning H , Likhacheva A , et al. Craniospinal irradiation with spinal IMRT to improve target homogeneity. Int J Rad Oncol Biol Phys. 2007;68(5):1402–09. 10.1016/j.ijrobp.2007.02.037 PMC1994073 17467921

[8] Parker W , Filion E , Roberge D , Freeman CR . Intensity‐modulated radiotherapy for craniospinal irradiation: target volume considerations, dose constraints, and competing risks. Int J Rad Oncol Biol Phys. 2007;69(1):251–57. 10.1016/j.ijrobp.2007.04.052 17707279

[9] Penagaricano JA , Papanikolaou N , Yan Y , Ratanatharathorn V . Application of intensity‐modulated radiation therapy for pediatric malignancies. Med Dosim. 2004;29(4):247–53. 15528065 10.1016/j.meddos.2004.04.007

[10] Lee CT , Bilton SD , Famiglietti RM , et al. Treatment planning with protons for pediatric retinoblastoma, medulloblastoma, and pelvic sarcoma: how do protons compare with other conformal techniques? Int J Rad Oncol Biol Phys. 2005;63(2):362–72. 10.1016/j.ijrobp.2005.01.060 16168831

[11] Bauman G , Yartsev S , Coad T , Fisher B , Kron T . Helical tomotherapy for craniospinal radiation. Br J Radiol. 2005;78(930):548–52. 15900062 10.1259/bjr/53491625

[12] Michalski JM , Klein EE , Gerber R . Method to plan, administer, and verify supine craniospinal irradiation. J Appl Clin Med Phys. 2002;3(4):310–16. 12383051 10.1120/jacmp.v3i4.2555 PMC5724535

[13] Hawkins RB . A simple method of radiation treatment of craniospinal fields with patient supine. Int J Rad Oncol Biol Phys. 2001;49(1):261–64. 10.1016/s0360-3016(00)01367-5 11163523

[14] Munshi A , Jalali R . A simple technique of supine craniospinal irradiation. Med Dosim. 2008;33(1):1–5. 18262116 10.1016/j.meddos.2007.03.004

[15] Parker WA , Freeman C . A simple technique for craniospinal radiotherapy in the supine position. Radiother Oncol. 2006;78(2):217–22. 16330119 10.1016/j.radonc.2005.11.009

[16] Thomadsen B , Mehta M , Howard S , Das R . Craniospinal treatment with the patient supine. Med Dosim. 2003;28(1):35–38. 12747617 10.1016/S0958-3947(02)00239-X

[17] Liu AK , Thornton D , Backus J , Dzingle W , Stoehr S , Newman F . Supine craniospinal irradiation setup with two spine fields. Med Dosim. 2009;34(3):214–16. 19647631 10.1016/j.meddos.2008.08.009

